# Role of surgery in patients with focally progressive gastrointestinal stromal tumors resistant to imatinib

**DOI:** 10.1038/srep22840

**Published:** 2016-03-07

**Authors:** Xiaodong Gao, Anwei Xue, Yong Fang, Ping Shu, Jiaqian Ling, Jing Qin, Yingyong Hou, Kuntang Shen, Yihong Sun, Xinyu Qin

**Affiliations:** 1 Department of General Surgery, Zhongshan Hospital, Fudan University, Shanghai, 200032, China; 2 Institute of General Surgery, Fudan University, Shanghai, 200032, China; 3 Department of Pathology, Zhongshan Hospital, Fudan University, Shanghai, 200032, China

## Abstract

The benefits of surgery for focally progressive gastrointestinal stromal tumor (GIST) during imatinib therapy are still in discussion. The aim of this study was to compare the outcomes of surgical resection of progressive lesions following tyrosine kinase inhibitor (TKI) therapy (S group) or TKI therapy alone (NS group) in GIST patients. We retrospectively investigated 57 patients with focally progressive GIST during imatinib therapy who were treated in Zhongshan hospital, Fudan University. Progression-free survival (PFS) and overall survival (OS) in the S group were significantly longer than those in the NS group. Among S group, the patients with R0 resection showed longer PFS than R2 resection; however, no difference was found between these two groups. Moreover, PFS and OS were not different in the NS-S group compared with S group. On multivariate analysis, surgery is an independent prognostic factor for longer PFS and OS. Our study supports the decision of treating GIST patients who were focally resistant to imatinib with surgery resection based on its benefit.

Gastrointestinal stromal tumors (GISTs) are the most common sarcomas of the gastrointestinal tract and are characterized by constitutive activation of the KIT or PDGFRA receptor tyrosine kinases. The crude annual incidence of clinically detected GISTs is about ten cases per million in Europe^1^ and could be higher in East Asia^2^. Localized GISTs represent a potentially curable disease if complete resection can be achieved. However, GISTs are associated with a high risk of recurrence and approximately 40–50% of patients with potentially curative resections develop recurrent or metastatic disease^3^,^4^. Moreover, many GISTs were unresectable or metastatic at the time of diagnosis. For those patients, imatinib is the primary therapeutic option, as its use is associated with an improved overall survival compared historic control^5^. The majority of GISTs are imatinib-sensitive at diagnosis, but during treatment they may acquire mutations in other exons that make them resistant to imatinib. The median progression-free survival (PFS) with imatinib therapy is 18–24 months^6^. Resistance can occur within a focal area of tumor which has previously regressed or remained stable, or it may be muti-focal and widespread in several metastatic deposit sites. Resection remains an option for patients with metastatic GISTs. Surgery of residual disease upon best clinical response conveys a survival benefit compared with historical controls in similar patient collectives treated with imatinib alone^7^. But in those that develop widespread progression of metastatic disease after imatinib treatment surgical resection is futile^8^. The benefits of surgery for focally progressive tumor after imatinib therapy are still in discussion. Some reports have demonstrated that resection of focally progressive disease can prolong survival and is not associated with early morbidity^9^,^10^. But not all studies have found resection of focally progressive disease to be of benefit. Mussi^7^ *et al*. compared patients undergoing resection of GIST masses at best clinical response to imatinib with focal progression, the later was found to have a significantly lower PFS.

Therefore, the aim of this study was to compare the clinical outcomes of combining surgery with TKI treatment to TKI treatment alone in patients with focally progressive GISTs who were initially responsive to imatinib.

## Results

### Patient characteristics

The baseline characteristics of GISTs in the S and NS groups are described in Table 1. The NS group patients were older than S group patients (median age 61 *vs*. 53 years; *p* = 0.039). And the NS group patients included more women (*p* = 0.031). Surgical interventions were described in Table 1S. The 30-day postoperative complication rate was 18.4%, including wound infection (4), abscess (2). There were no perioperative deaths.

### Survival outcomes

**Figure 1:**
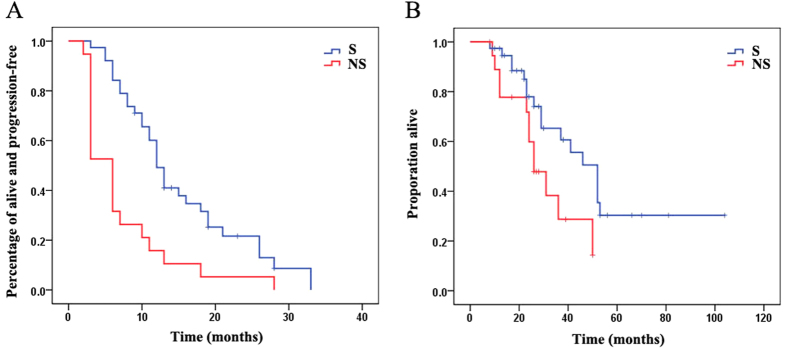
Progression-free survival (**A**) and overall survival (**B**) in the surgery group (blue line S group) versus the tyrosine kinase inhibitor group (red line) of gastrointestinal stromal tumor patients.

Over a median follow-up duration of 26 months (range 8–104 months), the median PFS for the S groups and NS groups were 12 months (95% CI 10.3–13.7 months) and 6 months (95% CI 4.7–7.3 months), respectly, *p* < 0.001. The median OS for the S group and NS group were 52 months (95% CI 42.0–63.0 months) and 26 months (95% CI 18.3–33.7 months). The long rank test of the difference was at *p* = 0.003, as show in Fig. 1.

Among S group patients, the patients with R0 resection showed a trend of longer PFS compared with the patients with R2 resection (median PFS 33 *vs.* 12months, *p* = 0.006). However, OS was not different according to the outcome of surgery (median OSs in both groups were not reached; *p* = 0.085), as show in supplementary Fig. 1.

**Figure 2:**
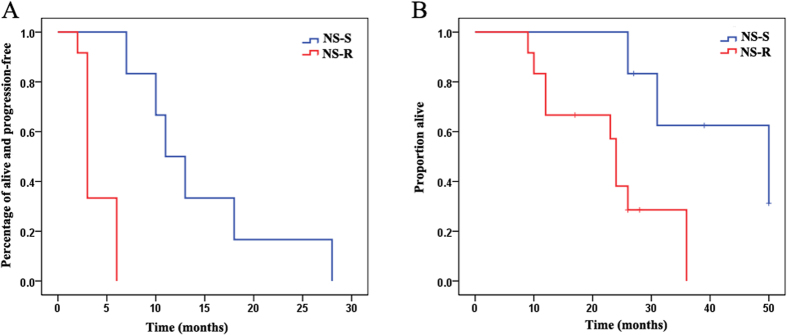
Progression-free survival (**A**) and overall survival (**B**) in the drug sensitive group (blue line NS-S group) versus the drug resistant group (red line NS-R) of gastrointestinal stromal tumor patients without surgery.

NS group patients generally received systemic therapy with escalation of imatinib dose or other TKIs. Among NS group patients, 6 patients (31.6%, NS-S) were sensitive to the drugs, 13 patients (68.4%, NS-R) were resistant to TKIs. PFS was significantly longer in drug sensitive patients (11 *vs.* 3 months; *p* < 0.001). OS was also significantly longer in these patients (54 *vs.* 24 months; *p* = 0.018), as show in Fig. 2. The PFS and OS of NS-S group patients won’t different from S group patients (PFS: 12 *vs.* 11months, *p* = 0.789; OS: 52 *vs.* 50 months; *p* = 0.949).

### Univariate and multivariate analysis of Progression-free survival and Overall survival

The results of the univariate and multivariate analyses for the PFS and OS outcomes using Cox regression modeling are list in Tables 2 and 3. Univariate analysis revealed that without peritoneum metastasis and surgery are associated with a longer PFS. And only surgery was associated with a longer OS. On multivariate analysis, surgery is an independent prognostic factor for longer PFS (HR 2.248, 95% CI 1.192–4.238, *p* = 0.012) and OS (HR 3.319, 95% CI 1.320–8.347, *p* = 0.011).

## Discussion

The resistance to imatinib is a life-threatening problem for patients with advanced GIST, because of the limited therapeutic strategies available. The rational for surgical resection of a focally progressive GIST lesion is to resect tumor that has gained resistance to TKI agents to stop disease progression. In our study, the PFS and OS were significantly longer for patients treated with surgery. These indicated that better treatment outcome when residual resistant disease had been resected.

There are little research studies about the role of surgery in patients with locally progressive GISTs. Al-Batran *et al*.^11^ demonstrated that patients with advanced GIST exhibiting focal disease progression during imatinib therapy may benefit from surgical resection. The median PFS was about 11 months. Hasegawa *et al*.^9^ indicated that surgical interventions in patients with GIST resistant to imatinib therapy are efficacious when complete resections are performed. Mussi *et al*.^7^ performed a retrospective analysis of 80 metastatic GIST patients who underwent surgery after imatinib. The 2-year postoperative PFS was significantly better in patients with best clinical response. However, the study didn’t show the benefit of surgery in local progression patients. All of these studies have presented low-level evidence because of lack of a control cohort of patients. To my knowledge, there has been no direct comparison between TKI therapy versus surgical resection of focally progressive GISTs combined with TKI therapy. In the study, we direct compared the clinical outcomes of combining surgery with TKI treatment to TKI treatment alone in local progressive patients.

Our results show a clear difference regarding PFS between patients who undergo surgery and not undergo surgery. The present report is the first retrospective study including a control group to show the benefits of surgery for local progressive patients. Other co-variables do not have a significant association with PFS in our multivariate analysis. And also our results indicated that surgery could significantly improve the survival of patients with local progression in univariate analysis and multivariate analysis. Most importantly, we did not find evidence that surgical procedures shortened life, as we did not observe surgery-related mortality. So we can consider surgery before switch to higher doses or second line therapies. We also found that there were no significant differences in PFS and OS between TKI sensitivity of patients and surgery of patients with local progression. It may indicate that if higher doses or another TKI was sensitive to the local imatinib-resistant disease, the operation could not be considered.

Concerning limitations of this study, the major problem with respective analysis is the bias in selecting patients. We found that sex and age were unbalanced between groups. We found that the patients were older in NS group. It indicated that older patients were apt to select TKI therapy only. In our study, both sex and age were not related to PFS or OS.

Although the best way to reduce or eliminate selection bias is a randomized controlled trial, such trials always had to be terminated early because of low participant accrual^12^,^13^. In the absence of randomized trials, only retrospective analysis can help physicians choose the best option for the patients. Our study supports the decision of treating GIST patients who were locally progression during imatinib therapy with surgery resection of resistant lesions based on its benefit. Especially, surgery resection of local resistant lesions may much more beneficial to the patients who were also resistant to higher doses imatinib or another TKI.

Surgical resection of local progressive lesion after TKI therapy is likely to be beneficial to patients with local recurrent or metastatic GISTs.

## Methods

### Patients

Between January 2005 and June 2014, a total of 98 patients were proven recurrent or metastatic GISTs who were treated with imatinib at Zhongshan Hospital Fudan University, Shanghai, China. Only patients who achieved a focally progression for more than 6 months following imatinib treatment according to the Response Evaluation Criteria in Solid Tumors (RECIS^14^) were included. Informed written consent was obtained from each participating patient. Progression was defined as a 20% increase in measurable lesion size (RECIST criteria), appearance of new lesion, or reappearance of a lesion that initially showed a complete response. Focally progressive disease was diagnosed by the presence of singular GIST lesion with an absolute growth in size on computed tomography (CT) in patients with previous disease responsive to imatinib. A total of 62 patients were histologically diagnosed with focally progressive GIST during the specified period, but 5 patients were excluded because of insufficient clinical data, such as being lost to follow-up. A final cohort of 57 patients was registered for the study, of whom 38 had undergone surgery after imatinib therapy (Group S) and of whom 19 had received treatment with TKI only (Group NS), as show in supplementary Fig. 2.

The response to TKI was mainly assessed according to the RECIST with CT scans, which were repeated at 2, 4, and 6 months after surgery or starting new TKI therapy method, and then every 3 months thereafter. Baseline evaluations included physical examinations, laboratory tests, chest X-ray, and CT of abdomen/pelvis with or without chest CT.

Survival was calculated from the initiation of higher dose of imatinib (600 mg/d) or sunitinib treatment or the initiation of surgery until death or the last follow-up. PFS was calculated from the initiation of higher dose of imatinib (600 mg/d) or sunitinib treatment or the initiation of surgery until disease progression. The last patient status was done in Jan 2015. Resection was considered complete if the entire gross tumor was removed with negative resection margins (R0 resection), and incomplete resection was defined as the presence of any gross residual tumors (R2 resection) or microscopic tumor in the surgical resection margin (R1 resection).

The study was approved by the ethics committees of Zhongshan Hospital, Fudan University. The methods were carried out in accordance with the approved guideline.

### Statistics

Baseline characteristics were compared using the Wilcoxon rank test for continuous variables (age) and χ^2^ test for categorical variables. PFS and overall survival (OS) were analyzed using the Kaplan-Meier method and compared using the log-rank test. The association of relevant clinical prognostic factors with PFS and OS was assessed using Cox proportional hazards regression modeling; the prognostic power of covariates was expressed by calculating hazard ratios (HRs) with 95% confidence intervals (CI). Variables with significance defined by *p* < 0.5 in the univariate model were included in the multivariate model, and adjusted HR with 95% CIs were calculated. *P* value < 0.05 was considered statistically significant. Analysis was performed with SPSS17.0 statistical software.

## Additional Information

**How to cite this article**: Gao, X. *et al*. Role of surgery in patients with focally progressive gastrointestinal stromal tumors resistant to imatinib. *Sci. Rep.* **6**, 22840; doi: 10.1038/srep22840 (2016).

## Supplementary Material

Supplementary Information

## Figures and Tables

**Table 1:** Characteristics of patients with focally progressive gastrointestinal stromal tumors resistant to imatinib therapy with surgery or without surgery.

Characteristics		Group S n = 38	Group NS n = 19	*P* value*
Age (year)				**0.039§**
	Median	53	61	
	(range)	(24–75)	(37–77)	
Gender				**0.031**
	Male	31 (41)	10 (41)	
	Female	7 (16)	9 (16)	
Primary tumor site				0.829
	Stomach	10 (16)	6 (16)	
	Small bowel	22 (33)	11 (33)	
	Others	6 (8)	2 (8)	
Cell type				0.878
	Spindle	35 (52)	17 (52)	
	Epithelioid	2 (3)	1 (3)	
	Mixed	1 (2)	1 (2)	
Tumor mitotic count per 50 HPFs				0.959
	≤5	4 (6)	2 (6)	
	6~10	5 (7)	2 (7)	
	>10	29 (44)	15 (44)	
Performance status (ECOG)				0.667
	0	30 (43)	13 (43)	
	1	7 (12)	5 (12)	
	2	1 (2)	1 (2)	
Gene type of primary tumors				0.783
	Exon 11	27 (41)	14 (41)	
	Exon 9	5 (6)	1 (6)	
	Wide type	1 (2)	1 (2)	
	Not availiable	5 (8)	3 (8)	
Site of tumor involvement				0.625
	liver	21 (31)	10 (31)	
	peritoneum	24 (39)	15 (39)	

HPF, high-power field.

*χ^2^ test, except ^§^Wilcoxon rank test.

**Table 2:** Univariate and multivariate analyses for progression-free survival in patients with focally progressive gastrointestinal stromal tumors resistant to imatinib therapy.

	PFS			
	Univariate analysis		Multivariate analysis	
	HR (95%CI)	*P* value	HR (95%CI)	*P* value
Age (year)	0.832(0.451–1.532)	0.554		
≥60				
<60				
Gender	1.379(0.743–2.559)	0.309	1.312(0.657–2.619)	0.441
Male				
Female				
Performance status	1.374(0.729–2.589)	0.325	0.827(0.396–1.728)	0.614
ECOG				
0				
1/2				
Primary tumor site	1.551(0.824–2.917)	0.174	1.417(0.685–2.931)	0.347
Gastric				
Nongastric				
Cell type	0.829(0.326–2.105)	0.693		
Spindle				
Nonspindle				
Tumor mitotic count per 50 HPFs	1.866(0.924–3.769)	0.082	1.552(0.695–3.464)	0.283
≤10				
>10				
KIT mutations.	0.747(0.311–1.794)	0.515		
Exon 11				
Others				
Site of tumor involvement Liver	0.725(0.415–1.267)	0.259	1.169(0.603–2.265)	0.644
Yes				
No				
**Peritoneum**	2.028(1.055–3.901)	**0.034**	1.706(0.741–3.929)	0.209
Yes				
No				
**Group**	2.615(1.457–4.694)	**0.001**	2.248(1.192–4.238)	**0.012**
S				
NS				

**Table 3:** Univariate and multivariate analyses for overall survival in patients with focally progressive gastrointestinal stromal tumors resistant to imatinib therapy.

	OS			
	Univariate analysis		Multivariate analysis	
	HR (95%CI)	*P* value	HR (95%CI)	*P* value
Age (year)	0.521(0.221–1.227)	0.136	0.392(0.146–1.048)	0.062
≥60				
<60				
Gender	1.325(0.571–3.074)	0.512		
Male				
Female				
Performance status	1.786(0.828–3.853)	0.139	0.951(0.377–2.402)	0.951
ECOG				
0				
1/2				
Primary tumor site	1.685(0.716–3.970)	0.232	1.310(0.480–3.577)	0.598
Gastric				
Nongastric				
Cell type	0.738(0.175–3.109)	0.679		
Spindle				
Nonspindle				
Tumor mitotic count per 50 HPFs	3.869(0.918–16.300)	0.065	2.482(0.529–11.648)	0.249
≤10				
>10				
KIT mutations	0.698(0.206–2.362)	0.563		
Exon 11				
Others				
Site of tumor involvement Liver	0.706(0.336–1.486)	0.359	0.820(0.369–1.823)	0.627
Yes				
No				
Peritoneum.	1.902(0.771–4.687)	0.163	1.211(0.433–3.388)	0.716
Yes				
No				
Group	2.329(1.049–5.170	**0.038**	3.319(1.320–8.347)	**0.011**
S				
NS				
