# Supplementary material for: Role of surgery in patients with focally progressive gastrointestinal stromal tumors resistant to imatinib
Source: Sci Rep. 2016 Mar 7;6:22840. doi: 10.1038/srep22840 (PMC4780000; doi:10.1038/srep22840)
Supplement: Supplementary Information [file srep22840-s1.doc]

**Role of surgery in patients with focally progressive gastrointestinal stromal tumors resistant to imatinib**

Xiaodong Gao1,2#, Anwei Xue1,2#, Yong Fang1,2, Ping Shu1,2, Jiaqian Ling1,2, Jing Qin1,2, Yingyong Hou3*, Kuntang Shen1,2*, Yihong Sun1,2, Xinyu Qin1,2

1 Department of General Surgery, Zhongshan Hospital, Fudan University, Shanghai, 200032, China

2 Institute of General Surgery, Fudan University, Shanghai, 200032, China

3 Department of Pathology, Zhongshan Hospital, Fudan University, Shanghai, 200032, China

**Supplementary TABLE 1** Description of surgery

| Group S | | No. of patients (%) |
| --- | --- | --- |
| Type of surgery | Hepatic resection  Bowel resection  Peritoneal mass resection  Colon resection  Rectal resection  Bladder resection | 12 ( 31.6% )  9 ( 23.7% )  24 ( 63.2% )  3 ( 7.9% )  1 ( 2.6% )  1 ( 2.6% ) |
|  |
| Outcome of surgery | R0 resection  R1 resection  R2 resection | 6 (15.8%)  0 (0)  32 (84.2%) |
| Number of resected organ | 1  2  More than two | 30 (78.9%)  6 (15.8%)  2 (5.3%) |
| Complication* | Grade 1  Grade 2  Grade 3 | 3 (7.9%)  3 (7.9%)  1 (2.6%) |

R0 absence of tumor in resection margin;

R1 microscopic presence of tumor in resection margin;

R2 presence of any gross residual tumors.

*Complication were classified according to accordion severity grading system.


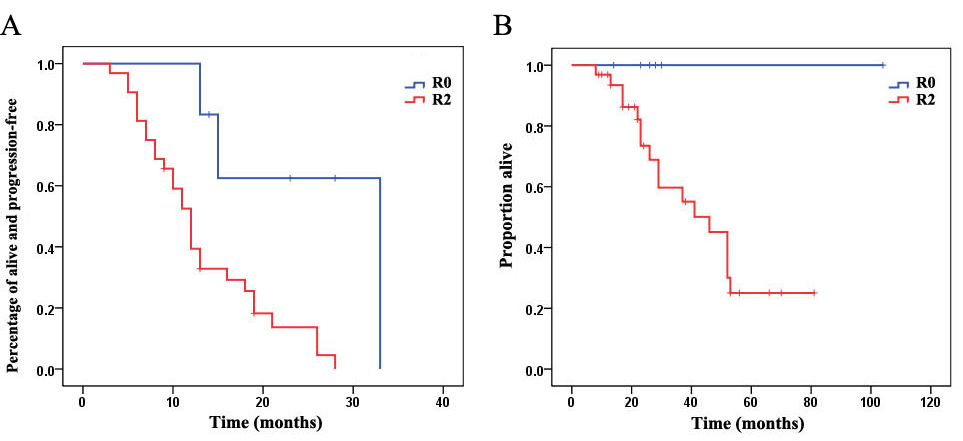


**Supplementary Fig.1** Progression-free survival (A) and overall survival (B) in the R0 surgery group (blue line R0 group) versus the R2 surgery group (red line R2 group) of gastrointestinal stromal tumor patients.


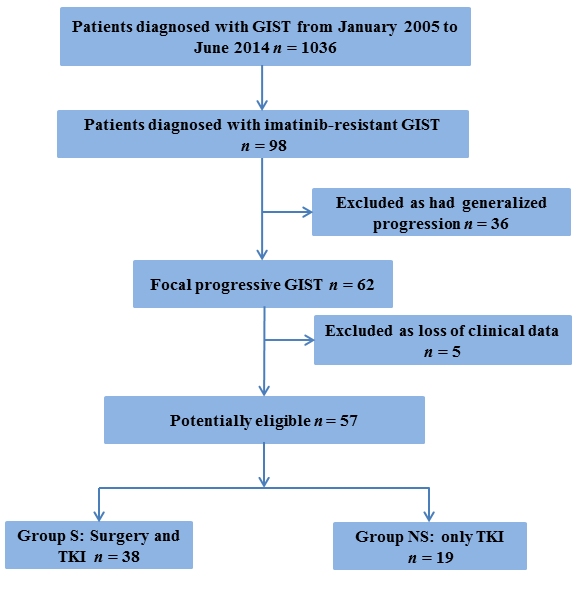


**Supplementary Fig.2** Flow chart of study design.
